# Anti-hyperglycaemic effects of herbal porridge made of *Scoparia dulcis* leaf extract in diabetics – a randomized crossover clinical trial

**DOI:** 10.1186/s12906-015-0935-6

**Published:** 2015-11-19

**Authors:** Senadheera Pathirannehelage Anuruddhika Subhashinie Senadheera, Sagarika Ekanayake, Chandanie Wanigatunge

**Affiliations:** Department of Biochemistry, Faculty of Medical Sciences, University of Sri Jayewardenepura, Nugegoda, Sri Lanka; Department of Pharmacology, Faculty of Medical Sciences, University of Sri Jayewardenepura, Nugegoda, Sri Lanka

**Keywords:** *Scoparia dulcis*, Leafy porridge, Anti-hyperglycaemic, Cholesterol

## Abstract

**Background:**

Leaf extracts of *Scoparia dulcis*, is used as a herbal remedy by diabetics worldwide. Fresh *Scoparia dulcis* porridge elicited a low glycaemic index (GI) and anti-hyperglycaemic effects when fed to diabetic Wistar rats. Commercially produced *Scoparia dulcis* porridge (SDC) elicited medium GI. Present study was aimed at studying the anti-diabetic effects of consumption of commercially produced *S. dulcis* porridge.

**Method:**

A randomized crossover clinical trial with type 2 diabetic patients (*n* = 35) on medication, with mild and moderate diabetes [fasting blood glucose (FBG) 126–300 mg/dL, age 35–70 years] was conducted. Within the first three months (study period 1) group 1 was the test and group 2 was the control. Following a wash-out period, the two groups were crossed over (study period 2: group 1 – control; group 2 - test). Test group consumed commercially produced SDC for 3 days/week for three months and the control group any other food. At the onset and end of each study period glucose measurements [Fasting Blood Glucose (FBG), HbA1c], lipid measurements (total cholesterol, HDL-C, LDL-C, triglycerides, cholesterol ratios), toxicity parameters (liver enzymes, creatinine, CRP, eGFR) were analyzed by enzyme assay kit methods using a KONELAB 20XT auto analyzer. Significances between groups were analyzed by one way ANOVA (normal distribution) and Mann Whitney test (if the values were not normally distributed). Within group comparisons were carried out by Bonferroni post hoc test.

**Results:**

During the crossover clinical trial HbA1c of group 1 decreased from 7.9 ± 0.5 to 6.5 ± 0.3 (*p* = 0.003) while HbA1c of group 2 decreased from 7.0 ± 0.3to 6.7 ± 0.3 while in the test group. Therefore, both test groups (1 and 2) elicited a decrease in HbA1c compared to respective control groups. Both test groups elicited a non significant decrease in FBG following the intervention (group 1 - from 174 ± 14 to 160 ± 10 mg/dL; group 2 - from 183 ± 13 to 160 ± 7 mg/dL). No significant differences (*p* >0.05) in insulin, cholesterol measurements (total cholesterol, LDL-C, HDL-C, triglycerides and cholesterol ratios) and atherogenic index between or within groups were observed. All other measurements (AST, ALT, ALP, creatinine, CRP, eGFR) were normal and not significantly different between or within groups.

**Conclusion:**

Porridge made with SDC leaf extract decreased FBG and HbA1c (*p* >0.05) of type 2 diabetic patients. The porridge had no effect on cholesterol measurements and no toxicity was observed at the dose tested. Therefore, the SDC porridge can be recommended as a suitable meal for diabetic patients.

## Background

The global exponential growth of diabetes has led to a concurrent rise in the usage of herbal remedies to treat diabetes due to their natural origin, free availability and lesser side effects [1]. Among the 21,000 herbs listed by the World Health Organization (WHO) as herbal remedies, only 150 species are utilized in preparation of large scale commercial herbal products [1]. *Scoparia dulcis* is used as a herbal remedy by diabetics worldwide with proven hypoglycaemic effects [2]. Products such as tablets and tea made from this herb are currently available in the international market [3, 4]. However, food products incorporating the herb are not thus far commercially available.

Rice based herbal porridges (*kola kenda*) are a common mode of ingesting herbal extracts in Sri Lanka. Herbal extracts used for these porridges have proven anti-hyperglycaemic and anti-hyperlipidaemic effects [2, 5, 6]. Porridges are more palatable and fulfilling compared to a mere water extract of herbs or tablets and could be ingested as a meal. A previous study with selected porridges proved that most of these porridges [rice: leaves: scraped coconut kernel in 25: 15: 10 (w/w/w) ratio] elicit low glycaemic index (GI) values in normal subjects [7]. A three month study with streptozotocin induced diabetic Wistar rats produced a significantly high (*p* <0.05) weight gain (opposing the catabolic action/ weight loss in diabetes), decreased HbA1c and diabetes related symptoms (polyuria, low activity rate etc.) in rats fed a diet made with *Scoparia dulcis* (SDC) porridge [8]. These effects were observed when the dried leaf solid dose was 200 mg/kgBW/day. Further, marked decreases in fasting blood glucose and urine sugar, with an increase in body weight had been observed in streptozotocin induced diabetic rats at a *Scoparia dulcis* extract dose of 250 mg/kgBW twice a day for 3 weeks [2]. Leaf extract of *S. dulcis* also increase binding of insulin to receptors, insulin synthesis and release of insulin from beta cells by stimulating Ca^2+^ influx [9]. Administration of *Scoparia dulcis* (200 mg/kgBW) extract to diabetic rats for 6 weeks, caused a significant reduction in serum and tissue cholesterol, triglycerides, free fatty acids, phospholipids, 3-hydroxy-3-methylglutaryl (HMG)-CoA reductase activity, very low-density lipoprotein and low-density lipoprotein cholesterol. The decreased serum high-density lipoprotein cholesterol, anti-atherogenic index, and HMG-CoA reductase activity in diabetic rats also returned to normal levels with the treatment [10]. The SDC porridge was developed as a commercial product targeting diabetic patients as ‘ready to use’ foods are gaining popularity among the urban populations where the prevalence of diabetes is high. The commercially produced porridge had the same ingredient ratio as the porridge used for previous studies on GI and diabetic Wistar rats and elicited medium GI in diabetics and in healthy adults due to the processing rice is subjected to during extrusion [7]. In the present study, the effects of long term consumption of commercially prepared *Scoparia dulcis* porridge on glycaemic, lipidaemic measurements and liver and kidney in type 2 diabetic patients were analyzed.

## Methods

### Preparation of the extruded meal and porridge

Tender leaves of *Scoparia dulcis* (15 kg), scraped coconut kernel (10 kg) and red rice [272 6B, Rice Research Institute, Bombuwala, Sri Lanka] (15 kg) were mixed and extruded at 110 –120 °C using JR 600 Extruder (InstraPor). Extruded meal was dehydrated using a hot air dryer (60–75 °C, 4–5 h). Dried meal was grinded using a Hammer mill (particle size 100 % pass through 0.5 mm mesh). Extruded meal was mixed in 1:1 ratio with boiled and dried intact rice grains [7]. Porridge was packed (40 g) in air tight packets which contained fresh leaves: rice: scraped coconut kernel in 13–15: 25–30: 10–13 (w/w/w) ratio.

### Clinical trial

A randomized crossover study was conducted in the Department of Biochemistry, Faculty of Medical Sciences, University of Sri Jayewardenepura, Sri Lanka. Patients were recruited from the Diabetes clinic of the Family Practice Centre, University of Sri Jayewardenepura. Minimum required sample size was 35 in each group. Sample size was calculated using *n* = 2 k σ^2^/Δ^2^ (comparing two means) by considering mean HbA1c change of 5 % from a mean of 8.0 (, with a SD of 0.6 (, k of 7.8 at 80 % power and 5 % two sided test (http://trials.slctr.lk/trials/104).

Type 2 diabetic patients, aged 35–70 years, with mild and moderate diabetes (FBG 126 – 300 mg/dL), who were not currently using *Scoparia dulcis* extract/ porridge were selected to participate in the study (Table 1).

**Table 1:** Demographic data of the study population

Number	35
Age	35–70 years
Mean age	53.6 ± 7.9 years
Disease	Type 2 diabetic patients [mild and moderate diabetes (FBG 126 – 300 mg/dL)]
Males	12
Females	23
Location	Colombo district
Weight	44 – 101.5 kg
Height	1.38 – 1.73 m
BMI	20 – 33
Waist circumference	79 – 128 cm
Waist circumference above normal	Females 100 %; males 64 %
Hip circumference	83 – 127 cm
W:H ratio above normal	Females 100 %; males 64 %
Total cholesterol	115 – 357 mg/dL
Fasting blood glucose	127 – 302 mg/dL
Obesity percentage	Females 87 %; males 58 %
Hypercholesterolaemia	61.5 %
Hypertriglyceridaemia	59.0 %

Patients with microvascular complications [diabetic retinopathy (background diabetic retinopathy/pre proliferative or proliferative retinopathy/maculopathy/mixed retinopathy), macular oedema, diabetic nephropathy (persistent albuminuria (>300 mg/day or >200 μg/min) that is confirmed on at least 2 occasions 3–6 months apart, progressive decline in the glomerular filtration rate (GFR), elevated arterial blood pressure (above 110–120/70–90 Hgmm), renal failure confirmed by a physician, diabetic neuropathy], macrovascular complications [patients who have experienced of a stroke or myocardial infarction or both at least once, patients who have been recommended for bypass surgeries, patients with non-healing wounds, gangrenes or amputated limbs/ toes], type 1 diabetics, who refused consent, and/or speak only Tamil were excluded from the study.

**Fig. 1:**
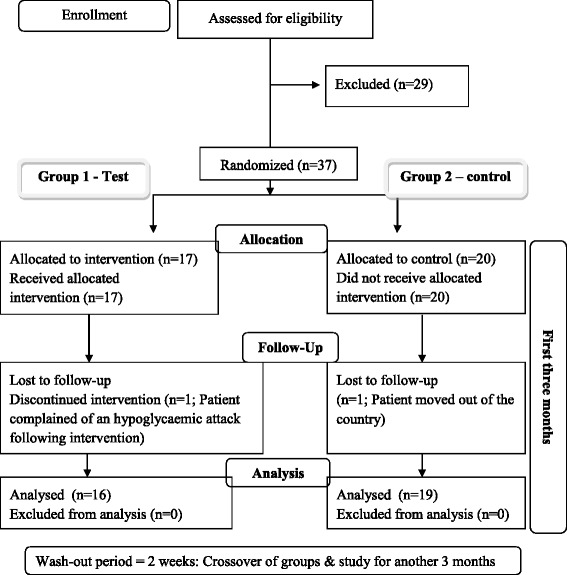
Flow diagram 1 study design

Study participants (*n* = 37) were grouped randomly in to test (group 1) (*Scoparia dulcis*) and control (group 2) groups (17–20/group) (every other patient from a list was included in to one group by the researchers). Each patient was in the test group for 3 months and in the control group for another 3 months. Test group was advised to consume one packet of *Scoparia dulcis* porridge once a day for 3 days/ week for 3 months and control group was advised to consume a normal breakfast except a green leafy porridge. One patient from the test group discontinued participation due to a hypoglycaemic attack subsequent to consuming the porridge. Following commencement of the study, one patient from the control group left the country. At the end of three months (study period 1) the groups were crossed over following a washout period of two weeks and the study was repeated for another three months (study period 2) with no dropouts. Thus the analyses included 35 patients (Flow diagram 1) (Fig. 1).

Blood (8 cc) were drawn from the median cubital vein of the patients (fasted for 8 – 10 h), by a trained phlebotomist, at the onset and end of each month during the study and serum was separated by centrifuging the samples at 3500 rpm. Liver enzymes (AST, ALT, ALP), creatinine, urea, CRP, lipid measurements (total cholesterol, LDL, HDL and TG) and blood glucose measurements (fasting blood glucose, HbA1c, insulin) were measured at the onset and at the end of the third month of the study. All parameters were measured by enzymatic kit assays (BIOLABO, France) except for plasma insulin which was measured by an ELISA assay (DRG Diagnostics Insulin assay kit). eGFR was calculated using the standard equation [186 x (creatinine/88.4)^-1.154^ x (Age)^-0.203^ x (0.742 if female) x (1.210 if black)]. All analyses were carried out on the same day that the blood samples were collected. Considering the cost and practical difficulties, the week 12 values of insulin and HbA1c were considered as unchanged and taken as the basal values for the second study period (week 14).

### Ethical approval

Ethical approval for the study was obtained from the Ethics Review Committee of the Faculty of Medical Sciences, University of Sri Jayewardenepura, Nugegoda (Approval No. 632/12). Written consent was obtained after explaining the study procedure to each volunteer prior to commencement of the study. The trial was registered with the Sri Lanka Clinical Trials Registry (SLCTR) (Registration No. SLCTR/2012/011).

### Statistical analysis

Significances of data of the clinical trial between groups were analyzed by one way ANOVA (normal distribution) and Mann Whitney test (if the values were not normally distributed). If the data do not violate the sphericity (Mauchly's test), significant differences between the means within groups at different time points were analyzed by one way repeated measures ANOVA. If the data violated sphericity, the presence of overall significant differences was identified by Greenhouse-Geisser correction. A *p* value <0.05 for Greenhouse-Geisser indicates a significant difference in means within groups. For such groups, within group comparisons were carried out by Bonferroni post hoc test to infer which means differed at different time points. Statistical analysis was carried out using SPSS version 16.

## Results

The study group consisted of 12 males and 23 females and mean age of participants was 53.6 ± 7.9 years. All the patients were residents of Colombo District, Western province, Sri Lanka. In the present study group 56 % and 34 % of patients have had diabetes for 1–10 years and more than 10 years respectively. Changes observed in mean glycaemic measurements of groups 1 and 2 during the study period are indicated in Table 2.

**Table 2:** Glycaemic parameters of groups 1 and 2

	Study period 1		Study period 2	
FBG (mg/dL)	Test (mean ± SEM)		Control (mean ± SEM)	
	0 week	12 weeks	14 weeks	26 weeks
Group 1^a^	174 ± 14	160 ± 10	199 ± 15	192 ± 19
	Control (mean ± SEM)		Test (mean ± SEM)	
	0 week	12 weeks	14 weeks	26 weeks
Group 2^b^	166 ± 11	171 ± 9	183 ± 13	160 ± 7
HbA1c (%)	Test (mean ± SEM)		Control (mean ± SEM)	
	0 week	12 weeks	14 weeks	26 weeks
Group 1^a^	7.9 ± 0.46	6.5 ± 0.30^a^	6.5 ± 0.30	7.1 ± 0.39
	Control (mean ± SEM)		Test (mean ± SEM)	
	0 week	12 weeks	14 weeks	26 weeks
Group 2^b^	7.6 ± 0.36	7.0 ± 0.31	7.0 ± 0.31	6.7 ± 0.24
Insulin (IU/L)	Test (mean ± SEM)		Control (mean ± SEM)	
	0 week	12 weeks	14 weeks	26 weeks
Group 1^a^	12.2 ± 10.4	14.9 ± 8.0	14.9 ± 8.0	12.1 ± 10.0
	Control (mean ± SEM)		Test (mean ± SEM)	
	0 week	12 weeks	14 weeks	26 weeks
Group 2^b^	16.6 ± 3.5	12.3 ± 1.5	12.3 ± 1.5	10.4 ± 1.2

Week 0 and week 14 indicates the basal serum parameters as those values have been obtained prior to the intervention

^a^ *n* = 16, ^b^ *n* = 19; SEM = standard error of mean; Superscripts indicate a significant difference in a parameter when compared to basal value within a study period (*p* <0.05)

When considering the two study periods [study period 1 (0–12^th^ week), study period 2 (14^th^–26^th^ week)], the initial (0 week and 14^th^ week) FBG between groups 1 and 2 were not significantly different (*p* = 0.63, *p* = 0.42). Although not significant, FBG in both test groups declined following the intervention (Table 2) when compared to the controls.

During the two study periods, initial (0 week and 14^th^ week) HbA1c between group 1 and 2 were not significantly different (*p* = 0.89, *p* = 0.31). However, a significant decrease (*p* = 0.003) in HbA1c in group 1 was observed during the intervention. In group 2 a significant decline in HbA1c was not observed (*p* = 0.98). Mean serum insulin levels were not significantly different (*p* >0.05) between or within group 1 and group 2 during both the study periods.

### Lipid measurements

Table 3 indicates the changes in serum cholesterol measurements of the two groups during the study period. There was no significant difference (*p* >0.05) in any of the lipid parameters (TC, LDL-C, TG and HDL-C) within groups 1 and 2 during both study periods. However, the TG in group 2 (control) was significantly higher when compared to group 1 (test) (*p* = 0.036) at the end of first 3 months (study period 1). A significantly high (*p* = 0.022) HDL-C level was observed in the test group (group 2) compared to the control (group 1) at the end of the second 3 months (study period 2). TC and LDL-C were above the upper limit of normal and TG was near the upper limit of normal in groups 1 and 2 during both study periods.

**Table 3:** Total cholesterol, triglycerides, LDL, HDL concentrations (mg/dL), total cholesterol: LDL, total cholesterol: HDL and LDL: HDL ratios of groups 1 and 2

	Study period 1		Study period 2	
TC (mg/dL)	Test (mean ± SEM)		Control (mean ± SEM)	
	0 week	12 weeks	14 weeks	26 weeks
Group 1^a^	228 ± 12	235 ± 13	246 ± 10	224 ± 10
	Control (mean ± SEM)		Test (mean ± SEM)	
	0 week	12 weeks	14 weeks	26 weeks
Group 2^b^	211 ± 14	233 ± 10	224 ± 14	232 ± 11
TG (mg/dL)	Test (mean ± SEM)		Control (mean ± SEM)	
Group 1^a^	0 week	12 weeks	14 weeks	26 weeks
	123 ± 9	139 ± 15	182 ± 20	175 ± 23
	Control (mean ± SEM)		Test (mean ± SEM)	
Group 2^b^	0 week	12 weeks	14 weeks	26 weeks
	142 ± 15	185 ± 15	171 ± 16	164 ± 19
LDL (mg/dL)	Test (mean ± SEM)		Control (mean ± SEM)	
Group 1^a^	0 week	12 weeks	14 weeks	26 weeks
	163 ± 11	168 ± 11	169 ± 10	151 ± 8
	Control (mean ± SEM)		Test (mean ± SEM)	
Group 2^b^	0 week	12 weeks	14 weeks	26 weeks
	137 ± 14	152 ± 9	147 ± 11	153 ± 10
HDL (mg/dL)	Test (mean ± SEM)		Control (mean ± SEM)	
Group 1^a^	0 week	12 weeks	14 weeks	26 weeks
	39.5 ± 2.1	39.0 ± 1.8	40.3 ± 1.9	37.3 ± 2.0
	Control (mean ± SEM)		Test (mean ± SEM)	
Group 2^b^	0 week	12 weeks	14 weeks	26 weeks
	43.9 ± 2.8	42.5 ± 2.1	42.8 ± 2.8	44.8 ± 2.3
	Study Period 1		Study Period 2	
TC:LDL	Test (mean ± SEM)		Control (mean ± SEM)	
	0 week	12 weeks	14 weeks	26 weeks
Group 1^a^	1.43 ± 0.04	1.49 ± 0.05	1.42 ± 0.03	1.47 ± 0.03
	Control (mean ± SEM)		Test (mean ± SEM)	
	0 week	12 weeks	14 weeks	26 weeks
Group 2^b^	1.63 ± 0.06	1.56 ± 0.03	1.56 ± 0.05	1.55 ± 0.04
TC:HDL	Test (mean ± SEM)		Control (mean ± SEM)	
	0 week	12 weeks	14 weeks	26 weeks
Group 1^a^	6.28 ± 0.40	5.69 ± 0.50	3.95 ± 0.28	4.20 ± 0.32
	Control (mean ± SEM)		Test (mean ± SEM)	
	0 week	12 weeks	14 weeks	26 weeks
Group 2^b^	5.22 ± 0.54	5.62 ± 0.34	5.50 ± 0.38	5.53 ± 0.43
LDL:HDL	Test (mean ± SEM)		Control (mean ± SEM)	
	0 week	12 weeks	14 weeks	26 weeks
Group 1^a^	4.33 ± 0.35	4.28 ± 0.28	6.29 ± 0.39	5.89 ± 0.37
	Control (mean ± SEM)		Test (mean ± SEM)	
	0 week	12 weeks	14 weeks	26 weeks
Group 2^b^	3.38 ± 0.48	3.69 ± 0.28	3.62 ± 0.32	3.71 ± 0.36
AI	Test (mean ± SEM)		Control (mean ± SEM)	
	0 week	12 weeks	14 weeks	16 weeks
Group 1^a^	0.49 ± 0.05	0.54 ± 0.06	0.61 ± 0.06	0.64 ± 0.7
	Control (mean ± SEM)		Test (mean ± SEM)	
	0 week	12 weeks	14 weeks	16 weeks
Group 2^b^	0.50 ± 0.06	0.62 ± 0.04	0.59 ± 0.05	0.54 ± 0.06

Week 0 and week 14 indicates the basal serum parameters as those values have been obtained prior to the intervention

^a^ *n* = 16, ^b^ *n* = 19; SEM = standard error of mean; Superscripts indicate a significant difference in a parameter when compared to basal value within a study period (*p* < 0.05)

Estimation of TC: HDL, also known as atherogenic or Castelli index and LDL: HDL are vital indicators of cardio vascular risk [11] as they indicate the imbalance between the cholesterol carried by atherogenic and protective lipoproteins. Total cholesterol and TG *per se* do not provide details on accurate risk levels, mainly in familial hypercholesterolaemia [12] (Real et al.). Therefore, to identify the risk level of the study population, total cholesterol: LDL, total cholesterol: HDL and LDL: HDL ratios of the groups 1 and 2 at study periods 1 and 2 were calculated (Table 3). However, no significant difference was observed between or within the groups 1 and 2 at any study period.

Although serum total cholesterol (TC) is normal, with low HDL level, the probability of development of atherosclerotic plaque is high [13]. As TC *per se* or HDL *per se* will not provide a clear picture regarding the risk of atherogenicity, atherogenic index (AI = log TC/HDL) was assessed. Atherogenic index of >0.21 indicates a high risk of atherosclerosis [14] (Table 3). However, no significant difference (*p* >0.05) between or within the groups 1 and 2 was observed for AI at any study period of the study.

### Toxicity measurements

Table 4 indicates the AST, ALT, ALP, creatinine, urea and CRP values in the test and control groups during the two study periods. A significant difference (*p* >0.05) was not observed between or within the two groups for all tested liver enzymes. Mean measurement variations of liver enzymes were within the normal reference range during both study periods (1 and 2). Mean eGFR of all study participants was within the normal range and was between 74.2 – 87.5 mL/min/1.73 m^2^, irrespective of the duration of diabetes (<1 year; 1–10 years; >10 years). No significant change was observed in urea, creatinine and CRP during both study periods.

**Table 4:** AST, ALT, ALP, creatinine, urea and CRP measurements of groups 1 and 2

	Study period 1		Study period 2	
AST(IU/L)	Test (mean ± SEM)		Control (mean ± SEM)	
	0 week	12 weeks	14 weeks	26 weeks
Group 1^a^	36.7 ± 4.7	37.0 ± 2.4	51.6 ± 5.6	41.4 ± 3.4
	Control (mean ± SEM)		Test (mean ± SEM)	
	0 week	12 weeks	14 weeks	26 weeks
Group 2^b^	32.5 ± 2.6	40.3 ± 4.3	39.0 ± 2.2	47.5 ± 4.1
ALT(IU/L)	Test (mean ± SEM)		Control (mean ± SEM)	
	0 week	12 weeks	14 weeks	26 weeks
Group 1^a^	43.3 ± 5.7	39.0 ± 3.7	55.5 ± 8.1	41.2 ± 8.9
	Control (mean ± SEM)		Test (mean ± SEM)	
	0 week	12 weeks	14 weeks	26 weeks
Group 2^b^	37.0 ± 4.7	42.6 ± 3.8	44.5 ± 5.3	43.0 ± 5.5
ALP(IU/L)	Test (mean ± SEM)		Control (mean ± SEM)	
	0 week	12 weeks	14 weeks	26 weeks
Group 1^a^	214 ± 19	231 ± 13^a^	232 ± 13	233 ± 12
	Control (mean ± SEM)		Test (mean ± SEM)	
	0 week	12 weeks	14 weeks	26 weeks
Group 2^b^	258 ± 14	329 ± 24^b^	330 ± 24	281 ± 20^c^
	Study period 1		Study period 2	
Creatinine (mg/dL)	Test (mean ± SEM)		Control (mean ± SEM)	
	0 week	12 weeks	14 weeks	26 weeks
Group 1^a^	0.85 ± 0.04	0.93 ± 0.06	1.00 ± 0.05	0.90 ± 0.05^a^
	Control (mean ± SEM)		Test (mean ± SEM)	
	0 week	12 weeks	14 weeks	26 weeks
Group 2^b^	0.75 ± 0.03	0.92 ± 0.03^b^	0.91 ± 0.03	0.91 ± 0.03
Urea (mmol/L)	Test (mean ± SEM)		Control (mean ± SEM)	
	0 week	12 weeks	14 weeks	26 weeks
Group 1^a^	4.2 ± 0.34	4.1 ± 0.55	5.5 ± 0.64	3.4 ± 0.28^a^
	Control (mean ± SEM)		Test (mean ± SEM)	
	0 week	12 weeks	14 weeks	26 weeks
Group 2^b^	4.5 ± 0.33	4.1 ± 0.24	4.2 ± 0.30	3.6 ± 0.18
CRP (mg/dL)	Test (mean ± SEM)		Control (mean ± SEM)	
	0 week	12 weeks	14 weeks	26 weeks
Group 1^a^	3.3 ± 0.74	3.4 ± 0.79	3.8 ± 1.05	3.0 ± 0.58
	Control (mean ± SEM)		Test (mean ± SEM)	
	0 week	12 weeks	14 weeks	26 weeks
Group 2^b^	4.3 ± 1.03	5.1 ± 0.88	5.1 ± 0.88	6.0 ± 1.11

Week 0 and week 14 indicates the basal serum parameters as those values have been obtained prior to the intervention

^a^ *n* = 16; ^b^ *n* = 19; SEM = standard error of mean; Superscripts indicate a significant difference in a parameter when compared to basal value within a study period (*p* < 0.05)

## Discussion

This clinical trial was designed to investigate the antidiabetic potential and toxic effects of consumption of a novel commercially produced herbal porridge in diabetics. The dried leaf solid dose in the porridge (per serving 35 mg/kgBW; 3 packets/week; 15 mg/kgBW/day) was 13–33 times lower than the doses (250 – 500 mg/kgBW) used in previously reported studies [2]. Despite the lower leaf dosage used in porridge, a reduction in FBG was observed in both test groups during study periods 1 (percentage reduction = 8 %) and 2 (percentage reduction = 13 %) compared to the two control groups. This indicates that even a low dose of *S. dulcis* leaves as used in this study could elicit significant anti-hyperglycaemic effects. A 1 % reduction of HbA1c over three months is known to reduce the risk of micro-vascular complications by 37 %, DM related complications and death by 21 % [15]. As there was a significant decrease (1.4 %; *p* = 0.003) in HbA1c in group 1 during the intervention and a non-significant (0.3 %; *p* = 0.98) decline in HbA1c in group 2, consumption of SDC porridge may contribute towards a reduction of micro-vascular and other fatal complications in diabetics [15]. Thus continuous consumption of *S. dulcis* porridge may help in reducing the morbidity and mortality due to diabetes.

A significant change in mean plasma insulin was not observed between or within the groups 1 and 2 indicating that there was no change in insulin synthesis, secretion or binding to receptors during the study period when the patients were in the control group. However, a decrease in FBG and HbA1c in both test groups during both study periods (study periods 1 and 2) was observed. This could be due to an increment of both the insulin level (synthesis and secretion) and binding to receptors mediated by SDC [9] or might be due to other mechanisms. Some mechanism attributed to consumption of *Scoparia dulcis* leaf extract include decreased oxidative stress in RINm5F pancreatic cells [16], increased glucose uptake by increased GLUT4 expression, translocation and activity [17], decreased glucose 6-phosphatase and fructose 1, 6-bisphosphatase enzymes [18] and increased hexokinase, glucose 6-phosphate dehydrogenase and glycogen synthase enzymes [18]. In this study a significant decrease in TC, TG and LDL-C concentrations were not observed as in previous studies [10], which could be due to the lower amount of active compounds in the porridge (35 mg/kgBW).

The study also revealed that 61.5 % and 59.0 % of the study population was hypercholesterolaemic and/or hypertriglyceridaemic respectively. More than 80 % of the T2DM patients in this study belonged to the high risk category according to the atherogenic index indicating a high susceptibility of these patients for cardio-vascular diseases.

An elevated occurrence in lipid abnormalities in patients who have had diabetes for more than one year was observed during this study. The percentages of hypercholesterolaemic and hypertriglyeridaemic diabetic patients were 82.7 % and 90.5 % respectively. Insulin resistance elevates the VLDL and apolipoprotein (apo) B-containing lipoproteins concurrently diminishing the suppressive effect of insulin on apo B secretion either by reducing apoB degradation or by inhibition of microsomal TG transfer protein activity [19]. This highlights the importance of regular assessment and appropriate goal directed management of lipid parameters once diabetes is diagnosed.

Absence of any significant elevation of AST, ALT, ALP, creatinine or urea or a decline in eGFR in both test groups compared to control during both study periods (study period 1 and 2), indicate that there is no acute or short term liver or kidney toxicity by consumption of the porridge at the dose given. Further studies are recommended to prove the effects of consumption of SDC porridge continuously for a long period.

CRP is an inflammatory marker and is generally elevated in diabetic patients [20]. As CRP is also an active participant of atherogenesis, there is a high risk of the occurrence of cardio-vascular diseases in diabetics [21]. There was no elevation of CRP in both the test groups during study period 1 and 2 compared to control. This suggests that SDC porridge does not contribute to elicit inflammatory reactions.

Precision of the results could have been increased with a higher number of patients, however due to the limited time period this could not be achieved. As the study was not a double blinded study and as the control group had not received a placebo, existence of bias to a certain extent in the present study is possible.

## Conclusion

Porridge made with *Scoparia dulcis* leaf extract reduce fasting blood glucose and HbA1c levels without elevating liver enzymes, creatinine, urea and CRP in type 2 diabetic patients. As a rice based herbal porridge is more palatable and fulfilling than a leaf extract or a tablet, the porridge used for this study would be a better breakfast meal for diabetics due to its medium GI [7], anti-hyperglycaemic effects and absence of toxicity. Diabetic patients should also be made aware regarding the importance of controlling their lipid parameters.

## References

[1] Modak M, Dixit P, Londhe J, Ghaskadbi S, Devasagayam TPA (2007). Indian herbs and herbal drugs used for the treatment of diabetes. J Clin Biochem Nutr.

[2] Das H, Chakraborty U (2011). Anti-hyperglycemic effect of *Scoparia dulcis* in streptozotocin induced diabetes. Res J Pharm Biol Chem Sci.

[3] Chawla R, Thakur P, Chowdhry A, Jaiswal S, Sharma A, Goel R (2013). Evidence based herbal drug standardization approach in coping with challenges of holistic management of diabetes: a dreadful lifestyle disorder of 21st century. J Diab Metabol Disord.

[4] Sweet Broomweed. Available at: http://www.drugs.com/npp/sweet-broomweed.html. Accessed on 23/11/2014.

[5] Choudhury RP, Garg AN (2007). Variation in essential, trace and toxic elemental contents in *Murraya koenigii* – A spice and medicinal herb from different Indian states. Food Chem.

[6] Veeramani C, Pushpavalli G, Pugalendi KV (2008). Antihyperglycaemic effect of *Cardiospermum halicacabum* Linn. leaf extract on STZ-induced diabetic rats. J Appl Biomed.

[7] Senadheera SPAS, Ekanayake S (2013). Development of a herbal leafy porridge from *Scoparia dulcis*. J. Agric. Sci.

[8] Senadheera SPAS, Ekanayake S, Wanigatunge C (2014). Anti-diabetic properties of rice based herbal porridges in diabetic Wistar rats. Phytother Res.

[9] Pari L, Latha M (2005). Antidiabetic effect of *Scoparia dulcis*: Effect of lipid peroxidation in streptozotocin diabetes. Gen Physiol Biophys.

[10] Pari L, Latha M (2006). Antihyperlipidemic effect of *Scoparia dulcis* (Sweet Broomweed) in streptozotocin diabetic rats. J. Med. Food.

[11] Millán J, Pintó X, Muñoz A, Zúñiga M, Rubiés-Prat J, Pallardo LF (2009). Lipoprotein ratios: Physiological significance and clinical usefulness in cardiovascular prevention. Vasc Health Risk Manag.

[12] Real JT, Chaves FJ, Martínez-Usó I, García-García AB, Ascaso JF, Carmena R (2001). Importance of HDL cholesterol levels and the total/ HDL cholesterol ratio as a risk factor for coronary heart disease in molecularly defined heterozygous familial hypercholesterolaemia. Eur Heart J.

[13] Dobiasova M (2004). Atherogenic Index of Plasma [Log (Triglycerides/HDL-Cholesterol)]: Theoretical and Practical Implications. Clin Chem.

[14] Shanmugapriya V, Mohanty PK, Anil KD (2013). Association between body mass index, lipid peroxidation and coronary lipid risk factors in hypothyroid subjects. Int J Med Sci Public Health.

[15] Stratton IM, Adler AI, Neil HAW, Matthews DR, Manley SE, Cull CA et al. Association of glycaemia with macrovascular and microvascular complications of type 2 diabetes (UKPDS 35): prospective observational study. Br Med J. 2000;321:405–12. doi:10.1136/bmj.321.7258.405. 10.1136/bmj.321.7258.405 PMC27454 10938048

[16] Latha M, Pari L, Sitasawad S, Bhonde R (2004). *Scoparia dulcis*, a traditional antidiabetic plant, protects against streptozotocin induced oxidative stress and apoptosis *in vitro* and *in vivo*. J Biochem Mol Toxic.

[17] Beh JE, Latip J, Abdullah MP, Ismail A, Hamid M (2010). *Scoparia dulcis* (SDF7) endowed with glucose uptake properties on L6 myotubes compared insulin. J Ethnopharmacol.

[18] Pari L, Latha M (2005). Antihyperglycaemic effect of *Scoparia dulcis*.: effect on key metabolic enzymes of carbohydrate metabolism in streptozotocin-induced diabetes. Pharm Biol.

[19] Bitzur R, Cohen H, Kamari Y, Shaish A, Harats D (2009). Triglycerides and HDL cholesterol stars or second leads in diabetes?. Diabetes Care.

[20] Hu FB, Meigs JB, Li TY, Rifai N, Manson JE (2004). Inflammatory markers and risk of developing type 2 diabetes in women. Diabetes.

[21] Mugabo Y, Li L, Renier G (2010). The connection between C-reactive protein (CRP) and diabetic vasculopathy. Focus on preclinical findings. Curr Diabetes Rev.

